# Standardisation is the key to the sustained, rapid and healthy development of stem cell‐based therapy

**DOI:** 10.1002/ctm2.1646

**Published:** 2024-04-04

**Authors:** Jing Zhang, Moran Suo, Jinzuo Wang, Xin Liu, Huagui Huang, Kaizhong Wang, Xiangyan Liu, Tianze Sun, Zhonghai Li, Jing Liu

**Affiliations:** ^1^ Department of Orthopedics First Affiliated Hospital of Dalian Medical University Dalian Liaoning Province China; ^2^ Key Laboratory of Molecular Mechanism for Repair and Remodeling of Orthopedic Diseases Dalian Liaoning Province China; ^3^ Stem Cell Clinical Research Center National Joint Engineering Laboratory First Affiliated Hospital of Dalian Medical University Dalian Liaoning Province China; ^4^ Dalian Innovation Institute of Stem Cell and Precision Medicine Dalian Liaoning Province China

**Keywords:** clinical application, regenerative medicine, standardisation, stem cell therapy

## Abstract

**Background:**

Stem cell‐based therapy (SCT) is an important component of regenerative therapy that brings hope to many patients. After decades of development, SCT has made significant progress in the research of various diseases, and the market size has also expanded significantly. The transition of SCT from small‐scale, customized experiments to routine clinical practice requires the assistance of standards. Many countries and international organizations around the world have developed corresponding SCT standards, which have effectively promoted the further development of the SCT industry.

**Methods:**

We conducted a comprehensive literature review to introduce the clinical application progress of SCT and focus on the development status of SCT standardization.

**Results:**

We first briefly introduced the types and characteristics of stem cells, and summarized the current clinical application and market development of SCT. Subsequently, we focused on the development status of SCT‐related standards as of now from three levels: the International Organization for Standardization (ISO), important international organizations, and national organizations. Finally, we provided perspectives and conclusions on the significance and challenges of SCT standardization.

**Conclusions:**

Standardization plays an important role in the sustained, rapid and healthy development of SCT.

## Graphical abstract

1. Stem cell therapy is developing rapidly and has made breakthrough progress.

2. Stem cell therapy standards mainly include three levels: ISO standards, international organisation standards and national standards, which complement and improve each other and jointly guide the standardisation of treatment.

3. The standardisation of stem cell therapy is not yet complete, and a complete standardisation system covering all aspects of stem cells is urgently needed.

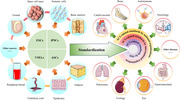

## 1 INTRODUCTION

With advances in medical technology and the improvement of quality of life, replacing or regenerating damaged tissue is becoming a reality. As an important part of regenerative medicine, stem cell‐based therapy (SCT) offers new hope to patients suffering from intractable diseases. The purpose of SCT therapy is to regenerate damaged cells and tissues within a human body using stem cells' unique characteristics or, in the case of replacing damaged cells with healthy cells, to deliver exogenous stem cells into the patient's body.^1^ In a biological system, stem cells are units of organisation responsible for the regeneration of different organs and tissues.^2^ In the human body, stem cells can be divided into five types according to their differentiation potential: totipotent, pluripotent, multipotent, oligopotent and unipotent stem cells.^3^ Totipotent stem cells have the highest potential and can differentiate into any of the three germ layers or form the placenta. The fertilised oocyte and the cells that divide during the first two divisions are totipotent stem cells.^3^ Pluripotent stem cells (PSCs) can form cells of the three germ layers, but cannot form cells of extraembryonic structures such as the placenta. One example is embryonic stem cells (ESCs) derived from blastocysts.^4^ Induced PSCs (iPSCs) are derived from somatic cells that have been reprogrammed into embryonic‐like states, which are also pluripotent.^4^, ^5^ In addition, the researchers found that adult tissues contain a population of small stem cells that also express pluripotency markers, naming them very small embryonic‐like stem cells (VSELs).^6^, ^7^ Multipotent cells can differentiate from a single germ layer into multiple specific cell types, the typical representative of which are mesenchymal stem cells (MSCs).^8^, ^9^ Oligopotent cells, such as common myeloid progenitor cells, can differentiate into only a few cell types depending on the tissue in which they reside.^10^ Unipotent cells have the lowest differentiation potential and can only differentiate along a single cell lineage, such as satellite cells of skeletal muscle.^11^ Stem cells used for SCT are capable of self‐renewal and differentiation into multiple cell lineages and can be of autologous, allogeneic or xenogeneic origin.^12^ Compared with differentiated cells, stem cells may be an ideal cell source for tissue regeneration because of their advantages such as easy to obtain in large quantities, high proliferation rate, and multidirectional differentiation.^2^, ^13^ Stem cells also have a paracrine function, indirectly playing a regeneration and repair role by releasing bioactive factors stored in exosomes.^14^, ^15^

**FIGURE 1:**
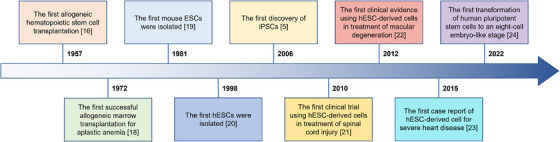
Important research progress that stem cell‐based therapy (SCT) has made over the decades. These public data are derived from literature searches on PubMed (https://pubmed.ncbi.nlm.nih.gov/). hESCs, human embryonic stem cells; iPSCs, induced pluripotent stem cells; SCT, stem cell‐based therapy.

The world's first recorded allogeneic haematopoietic stem cells (HSCs) transplantation for the treatment of leukaemia was completed in 1957.^16^ Since bone marrow transplantation was unknown at the time, all six patients in this study died and only two patients showed evidence of transient engraftment.^16^ This result indicated that the graft had failed. However, in the following two years, researchers discovered that bone marrow transplantation from healthy identical twins could be used to treat leukaemia.^17^ Since then, after decades of rapid development, many significant advances have been made the field of SCT, such as the discovery of ESCs and iPSCs, and their application in different diseases.^5^, ^18^, ^19^, ^20^, ^21^, ^22^, ^23^, ^24^ It has not only improved the confidence of researchers and patients in SCT, but also promoted the rapid development of SCT market (Figure 1).

SCT involves a complex and precise process, including multiple links such as tissue collection, stem cell isolation, purification, expansion, identification testing, preservation, recovery, and auxiliary material production.^25^ Irregularity in any of these links may have an impact on the therapeutic effect of SCT, and then affect the development of SCT. The rapid development of SCT presents challenges for regulatory authorities.^25^, ^26^, ^27^ Although SCT has broad application prospects, it has not yet achieved large‐scale clinical application. When things do not work as they should, it often means that standards are absent. Standards cover a wide range of activities, which may involve product manufacturing, process management and material supply.^28^ The transition of SCT from small‐scale, customised experiments to routine clinical practice requires standards to assist. Standardisation can not only help SCT develop safely, effectively and ethically, but also prevent its premature commercialisation.^28^, ^29^, ^30^ Therefore, many countries, regions and international organisations around the world have developed corresponding SCT standards, which will strongly promote the further development of the SCT industry. This article aims to summarise the clinical application progress of SCT, and focus on the research progress of SCT standardisation.

## 2 THE APPLICATION STATUS OF SCT

### 2.1 Types and characteristics of stem cells

Clinically, there are many stem cells used for treatment in the human body, which can be divided into the following four types: ESCs, VSELs, iPSCs and adult stem cells (ASCs).^1^, ^2^, ^31^ These cells have distinct characteristics in SCT (Table 1).

**TABLE 1:** Types and characteristics of stem cells used in SCT.

Types	Potency	Main sources	Isolation/expansion	Advantages	Disadvantages	References
ESCs	Pluripotent	Inner cell mass	Challenging	High differentiation potential	Ethical issues, risk of immune rejection and teratoma formation	^32^–^38^
iPSCs	Pluripotent	Somatic cells	Challenging	May overcome ethical and immunogenicity issues	Risk of teratoma formation	^37^, ^39^–^45^
VSELs	Pluripotent	BM, UCB, mPB and gonads	Challenging	May overcome ethical and teratoma formation issues	Quiescence and limited number	^6^, ^7^, ^46^–^48^
ASCs	Multipotent, oligopotent, unipotent	MSCs, HSCs, NSCs and EPSCs	Most can be easily	Broad sources, may overcome ethical and tumourigenicity issues.	Low differentiation potential, risk of immune rejection	^12^, ^48^–^54^

Abbreviations: ASCs, adult stem cells; BM, bone marrow; EPSCs, epidermal stem cells; ESCs, embryonic stem cells; HSCs, haematopoietic stem cells; iPSCs, induced pluripotent stem cells; mPB, mobilised peripheral blood; MSC, mesenchymal stem cells; NSCs, neural stem cells; UCB, umbilical cord blood; VSELs, very small embryonic‐like stem cells.

ESCs are mainly obtained from the inner cell mass of blastocysts.^32^, ^33^ Although ESCs are pluripotent, ethical issues, immune rejection, and the risk of teratoma as stem cells serve as natural units of selection for tissue formation limit their clinical application.^34^, ^35^, ^36^, ^37^, ^38^ IPSCs can overcome the ethical and immunogenicity challenges of ESCs and are a promising alternative.^39^, ^40^, ^41^ However, iPSCs transplantation also carries the risk of teratoma formation due to the possibility of transplanting undifferentiated cells.^37^, ^42^ Therefore, during the stem cell treatment process, it is important to focus on the patient's health status to reduce the potential risks of stem cell treatment as much as possible. In addition, both ESCs and iPSCs face challenges in cell isolation and expansion.^43^, ^44^, ^45^ In contrast, VSELs have emerged as an attractive cell type. VSELs are PSCs involved in blood vessel and tissue regeneration, forming a pool of recruited stem/progenitor cells that play specific and important roles in organ repair.^6^, ^46^ In human bone marrow (BM), umbilical cord blood (UCB), mobilised peripheral blood (mPB), and gonads, VSELs have been observed to exist.^7^, ^47^ Nonetheless, the quiescence and limited number of VSELs make isolation of these cells challenging.^47^, ^48^ VSELs have limited ability to expand in vitro. In order to stimulate the proliferation of cells without compromising their pluripotency, we need to understand their biological properties.^47^ Currently, the commonly used cell types in SCT are ASCs, which mainly includes MSCs, haematopoietic stem cells (HSCs), neural stem cells (NSCs) and epidermal stem cells (EPSCs), covering multiple potential categories of multipotent, oligopotent and unipotent.^12^, ^48^, ^49^, ^50^ Among them, MSCs are the most widely used subtype, including BM, adipose, umbilical cord, amniotic membrane and other tissue sources.^50^, ^51^ ASCs have the characteristics of low immune rejection, and most ASCs are easy to isolate and expand.^50^, ^52^, ^53^ It should be noted that their insufficient differentiation potential prevents them from naturally crossing genetic barriers to differentiate into other lineages.^54^

Determining in vivo biodistribution after administration is an important research component in the development of SCT. Through different routes of administration, the biodistribution of stem cell preparations in the body changes, leading to possible changes in their therapeutic effects. Current research mainly focuses on analysing the biodistribution of MSCs in the body after administration. Intravenous access is the most common route for cell transplantation. Since MSCs are much larger in diameter than HSCs, the first obstacle after intravenous injection is the lung capillary bed.^55^, ^56^ Studies have found that stem cells transplanted through veins are mainly distributed in the lungs in the early stage, and then distributed in the spleen, liver and other organs.^57^, ^58^ Distribution of MSCs at usual bleeding sites was observed in patients with haemophilia A at 24 h.^59^ Unlike intravenous administration, intravenous administration bypasses the filtration of the lungs. Sood et al. injected labelled stem cells into the superior pancreaticoduodenal artery and splenic artery respectively through peripheral venous route or targeted route to explore appropriate administration methods for diabetic patients.^60^ The results show that the biodistribution of stem cells following arterial infusion is characterised by systemic delivery, lack of pulmonary capture, and selective homing and retention in the pancreas compared with the intravenous route.^60^ Interestingly, most of the cells migrated to the liver, spleen, and BM, with few remaining in the myocardium.^61^, ^62^, ^63^ Homing and interstitial migration are two principal modes of stem cell trafficking, which is defined as the oriented or directed movement of a cell towards a particular anatomic destination.^64^ This phenomenon involves chemical attractants, adhesion molecules, and specific pathways that guide migration to specific sites or niches.^64^, ^65^ By manipulating stem cell trafficking, the regeneration and repair effect of damaged tissue can be improved, and the cells may also serve as vehicles for drug delivery.^64^ Stem cells can not only be infused through blood vessels, but can also be injected directly into lesions. A clinical study showed that after injecting MSCs into the intervertebral disc, mesenchymal stem cells were found in the intervertebral disc with chondrocyte‐like differentiation 8 months later.^66^ After 28 months, these cells disappeared from the disc.^66^ Research on the in vivo biodistribution of stem cells injected into other diseased areas such as the reproductive and urinary systems, digestive systems, and the central nervous system is still at the animal model stage, and the results of different studies are different and need to be further studied.^56^ Various methods including physical, chemical, and genetic modification have been explored to improve the targeted delivery of stem cells to diseased tissues.^67^ At the same time, limitations to these methods should also be considered, as they may induce off‐target toxicity and other unintended effects on stem cells, as well as lead to transient expression of molecular targets.^67^, ^68^

In order to target stem cells to specific tissues, researchers have developed a variety of corresponding stem cell delivery devices. The most studied ones are devices that apply stem cells to the heart. There are three main ways to deliver stem cells to the heart: transendocardial delivery, intracoronary delivery, and transcoronary delivery.^69^ Devices used for transendocardial delivery of stem cells mainly include MyoCath catheter, MyoStar catheter, Helix catheter, C‐Cath catheter and Phoenix combination delivery system. Both the MyoCath catheter and the MyoStar catheter are integrated systems, meaning they are made up of a support catheter and a core. Unlike the MyoCath catheter's stainless‐steel needle, the MyoStar catheter has a nitinol needle. Such two catheter systems have been used in the field of cardiac regeneration and repair.^70^, ^71^, ^72^ Unlike the previous two catheter systems, the Helix catheter is a non‐integrated system that also allows safe transplantation of stem cells to improve myocardial contractility.^73^ The C‐Cath catheter is made of nickel‐titanium alloy, which improves stem cell retention in the myocardium and ensures infusion safety and ease of use.^74^ The Phoenix combined conveyor system is an emerging piece of equipment. Using this system to perform BM laser revascularisation can improve patients' living quality.^75^ Devices for transcoronary delivery of stem cells are typically percutaneous transluminal coronary angioplasty catheters. These balloon catheters include ‘monorail’ and ‘over‐the‐wire’ catheters, with the latter being more common and requiring a guiding catheter to reach its destination. The Maverick catheter and the Cricket micro‐infusion catheter are both over‐the‐wire balloon catheters that deliver stem cells.^76^, ^77^ A typical device for delivering stem cells through coronary arteries is the TransAccess catheter system, currently marketed by Philips under the name Pioneer. This catheter system is injected through transcoronary venous injection. Compared with other catheter systems, the Pioneer catheter system has higher stability, and the injection pressure is not as easy to cause ejection as ordinary catheter systems.^69^, ^78^

Applying stem cells to the central nervous system is something many clinicians aspire to achieve. Due to the large size of the damaged area of the human brain, it is challenging to complete treatment with a single cell injection. To reduce the number of punctures and increase the distribution of transplanted stem cells, Brecknell et al.^79^ developed the radial branch deployment (RBD) syringe system. The system is positioned at an angle to the guidance axis to allow stem cells to reach further targets and can also be rotated to avoid potential ‘hot spot’ effects.^80^ Subsequently, a team improved the device to meet clinical safety requirements in order to reduce the risk of accidents.^81^ Spinal injection also has a corresponding delivery platform, which can deliver stem cells to the spinal cord stably and safely.^82^, ^83^ Clinically, stem cells can also be loaded onto cell scaffolds and transplanted to other sites through endoscopic injection, which provides more possibilities for treatment options.

### 2.2 Clinical application status of SCT

**FIGURE 2:**
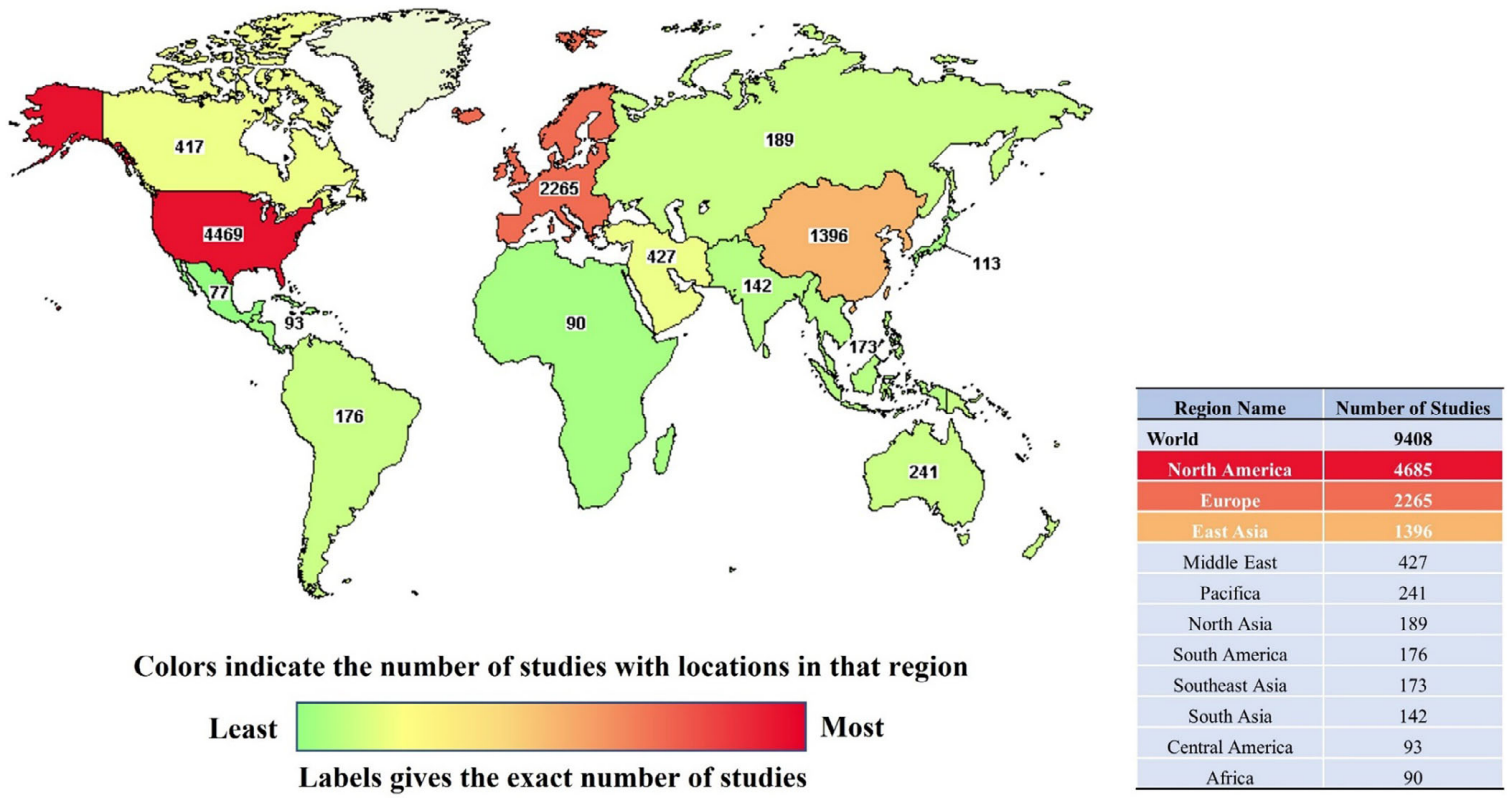
Number of stem cell‐based therapy (SCT) clinical trials registered on ClinicalTrials.gov by 2022. These public data, including basic information and numbers, are derived from stem cell‐related clinical trials registered on ClinicalTrials.gov as of December 2022 (https://classic.clinicaltrials.gov/). SCT, stem cell‐based therapy.

In order to speed up the clinical application of SCT, on the basis of the safety data from previous animal experiments, countries around the world have carried out extensive SCT clinical trials. As of December 2022, there have been over 9400 stem cell‐related trials registered in a clinical trial registration site (www.clinicaltrials.gov), most of which are mainly concentrated in North America, Europe and East Asia. Although not all registered clinical trials have been conducted or have results, these data still reflect the strong appeal of SCT to researchers from all over the world (Figure 2).

**FIGURE 3:**
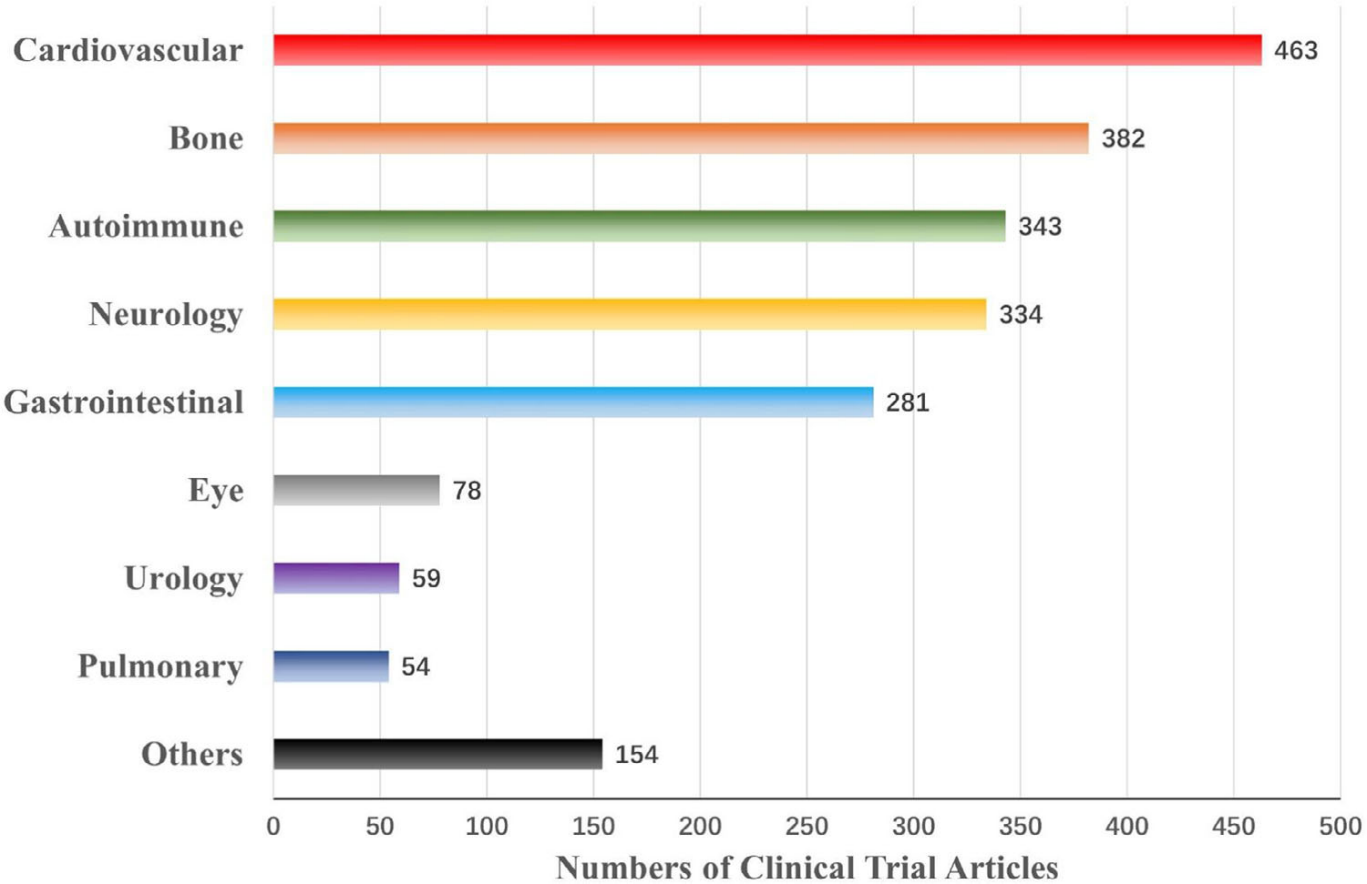
Number of stem cell clinical trial articles in different categories of diseases on StemCreDB by 2022. These public data, including disease categories and the number of clinical trial articles, are derived from statistics on the StemCreDB as of December 2022 (https://stemcredb.net/). SCT, stem cell‐based therapy; StemCreDB, stem cell clinical research database.

**FIGURE 4:**
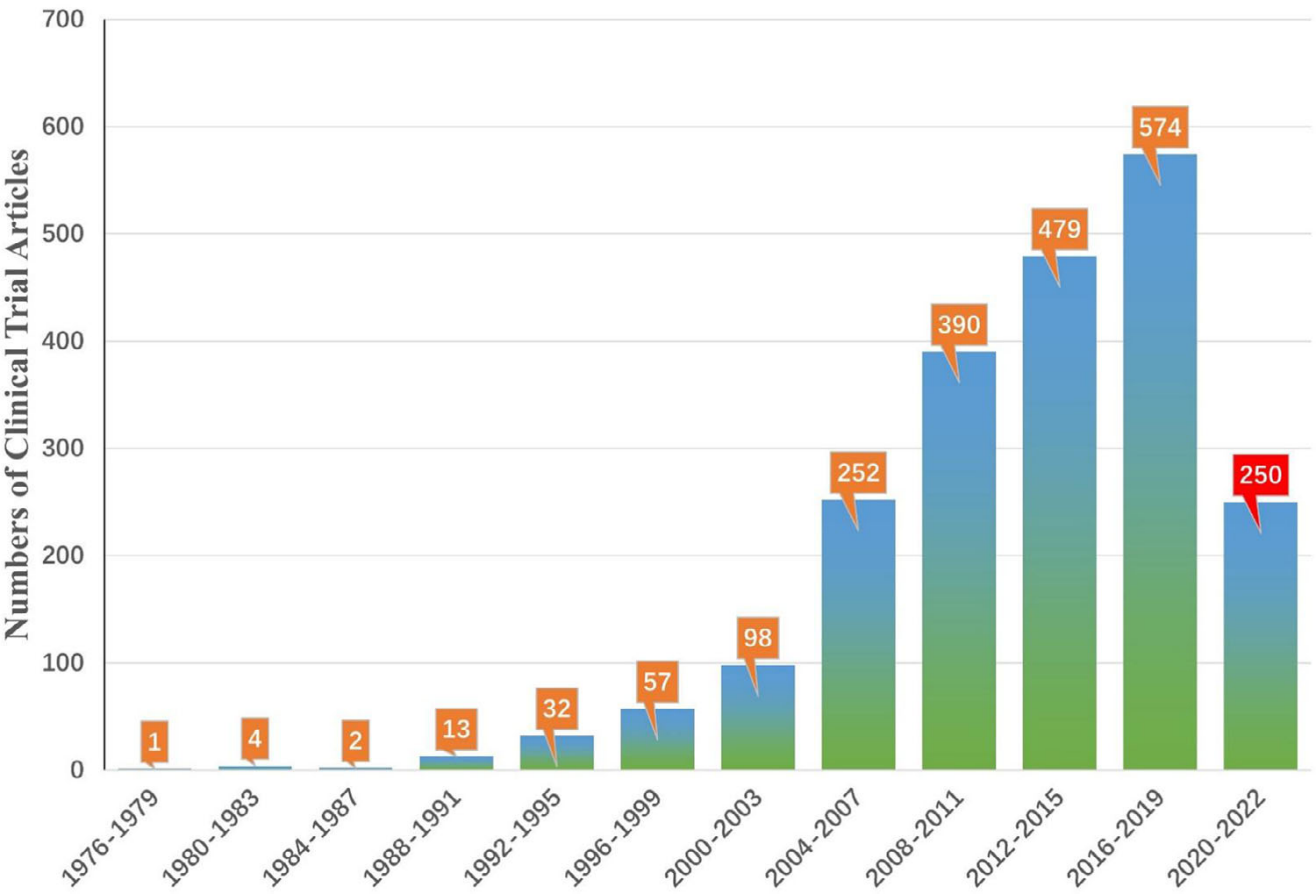
Number of stem cell clinical trial articles in different years StemCreDB by 2022. These public data, including the number of stem cell clinical trial articles every 4 years, are derived from statistics on the StemCreDB as of December 2022 (https://stemcredb.net/). SCT, stem cell‐based therapy; StemCreDB, stem cell clinical research database.

**FIGURE 5:**
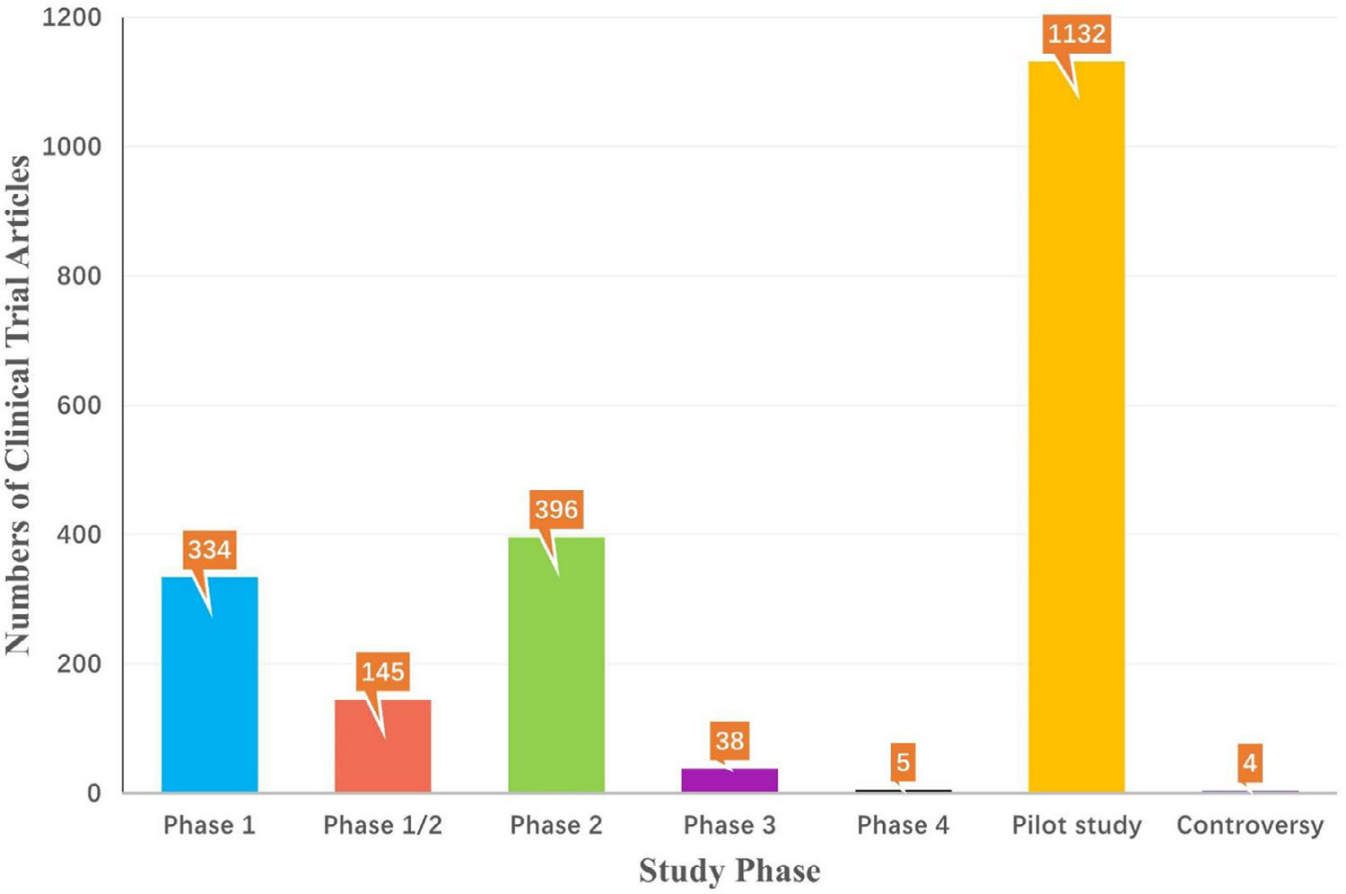
Number of stem cell clinical trial articles in different study phases on StemCreDB by 2022. These public data, including the number of stem cell clinical trial articles in different study phases, are derived from statistics on the StemCreDB as of December 2022 (https://stemcredb.net/). SCT, stem cell‐based therapy; StemCreDB, stem cell clinical research database.

Although SCT has not really achieved large‐scale clinical application, its clinical studies in multiple disease systems have been reported and counted. According to the statistics of the Stemcell Clinical Research Database (DB),^84^ the current clinical trials of SCT involve Cardiovascular, Skeletal, Nervous, Immune, Gastrointestinal, Pulmonary, Urinary, Ophthalmic and others. Among them, most of the SCT clinical trial articles focus on the first five categories, especially the largest number of studies on cardiovascular system diseases (Figure 3). Furthermore, from 1976 to the present, these clinical trials have shown an increasing trend. It should be noted that during the 3 years from 2020 to 2022, although the number of SCT clinical trial articles is not as high as the sum of the number of articles in the previous four years, the difference is not much different (Figure 4). Considering multiple factors such as immune rejection, ethical barriers and treatment costs, the transplanted cells are mainly autologous. In addition, most trials are still in the exploratory research stage, and the number of phase III and above clinical trials is relatively small (Figure 5). Therefore, there is still a certain distance for SCT to fully realise large‐scale clinical application.

Different types of stem cells have achieved different clinical translational research results. The application of ESCs is restricted by ethical considerations and strict supervision, and related research mainly uses ESCs‐derived cells. Since Schwartz et al.^22^ reported the first use of HeSC‐derived retinal pigment epithelium (RPE) to treat retinal diseases in 2012, multiple studies have demonstrated the safety and efficacy of ESC‐RPE in the treatment of patients with age‐related macular degeneration (AMD) and Stargardt's macular dystrophy.^85^, ^86^, ^87^, ^88^, ^89^ In order to quickly and efficiently generate RPE cells from ESCs, researchers have developed a method for rapid directional differentiation of cells.^90^ The described protocol can be used for clinical‐grade cell production.^90^ Furthermore, ESC‐derived cells have also shown potential therapeutic efficacy in diseases such as type 1 diabetes, meniscal damage and interstitial cystitis.^91^, ^92^, ^93^ Similar to ESCs, some scholars have also explored RPE cell sheets differentiated from iPSCs to treat AMD. One year later, the patients' best‐corrected visual acuity had not improved or worsened, and cystoid macular oedema was present.^94^ To overcome fundamental limitations of the donor‐derived MSC production process, Bloor et al.^95^ used iPSC‐derived MSCs to treat steroid‐resistant acute graft versus host disease (SR‐aGvHD) and found that the therapy was safe and tolerable. Interestingly, iPSCs can be used not only for treatment but also for disease modelling and drug research. IPSCs can be obtained from amyotrophic lateral sclerosis (ALS) patients with different genetic phenotypes, and the genetic background of the cells is not affected by the reprogramming process.^96^ Therefore, iPSCs can well assess the impact of ALS on patients and accelerate ALS in vitro modelling and drug development.^96^, ^97^ Notably, transplantation of iPSCs‐derived glial cells rather than derived motor neurons can treat ALS by improving the neuronal environment.^96^ In clinical practice, ASCs are the most studied. Researchers are harnessing the regenerative capabilities of ASCs to treat many diseases such as heart, brain, pancreatic and eye diseases. Currently, ASC is the gold standard for clinical application, especially represented by MSCs.^98^ MSCs have promising applications due to their ease of isolation and expansion, multilineage differentiation capacity, immune modulation, homing and migration, and few ethical constraints.^3^ ASC‐based therapies have a wide range of clinical applications, covering the treatment of diseases in cardiovascular, orthopaedic, autoimmune, neurological, metabolic and other systems.^3^, ^98^ As PSCs, VSELs constitute a recruitable stem cell pool and can also play an important role in tissue repair.^46^, ^99^ Studies have found that VSELs in patients with lung disease can be recruited into peripheral blood (PB) to participate in tissue repair.^46^ Recruitment of VSELs has also been detected in the PB of patients with critical limb ischaemia (CLI). These cells can differentiate towards the vascular lineage and may generate human endothelial lineage cells.^100^ Efforts are underway around the world to encourage the development of SCT, although this may change in the future.^101^

### 2.3 Market development status of SCT

Robust clinical trials have led to significant market growth, and the market size of SCT has grown rapidly. According to the SCT market report released by Global Market Insight in 2021, the global SCT market size was estimated at USD 8.9 billion in 2020, with North America having the largest market share at 41.5%, which may benefit from strong government support.^102^ The report also predicts that with the advancement of technology and increased attention for biologics, the market size of SCT will grow at a compound annual growth rate (CAGR) of more than 10.6% from 2021 to 2027.^102^, ^103^ However, the development of SCT also faces limiting factors. For example, ethical issues of PSCs and their inherent properties including tumourigenicity and immunogenicity limit their use.^4^, ^38^, ^104^, ^105^ Additionally,

inconsistencies in the stability, differentiation, and trafficking of MSCs, as well as differences between preclinical and clinical studies, pose challenges to the application of SCT.^106^, ^107^ All these challenges may adversely affect the market development of SCT.

## 3 THE DEVELOPMENT STATUS OF SCT‐RELATED STANDARDS

SCT has made remarkable achievements in both disease treatment and market size. However, SCT includes a complete and complicated process, involving multiple links such as cell collection, culture, transportation, and detection. Irregular operation in any link may affect the actual therapeutic effect of SCT. Therefore, establishing relevant standards for stem cell research and industry is crucial to the healthy development of SCT. Only in this way can a smooth and efficient assessment of stem cell products, production facilities, therapeutic efficacy and market trends be ensured based on sound scientific principles.

### 3.1 SCT‐related standards by ISO

At present, the international standardisation work is mainly carried out in the International Organization for Standardization (ISO). ISO is an international standard development organisation composed of standards bodies from 167 different countries. The standards it develops are recognised by international experts around the world. There are more than 250 technical committees (TC) under ISO, which develop most ISO standards. Among them, the TC involved in the development of SCT‐related standards is mainly ISO/TC 276 Biotechnology. ISO/TC 276 has published 26 standards, 12 of which are related to cell therapy. These standards include four aspects. First, these standards specify the requirements for biobanks of human BM‐derived MSCs (hBMSCs), human and mouse PSCs, and human UC‐derived MSCs (hUCMSCs), including the collection of biological source material and associated data, isolation, culture, characterisation, quality control, cryopreservation, storage, thawing, disposal, distribution and transport.^108^, ^109^, ^110^ It is worth noting that the document listing the various requirements and regulations for cultivating and using human and mouse PSCs is named ISO 24603, which is also the first stem cell related standard for the ISO.^109^ Second, standards provide general requirements for cells, including cell transport and cell counting, as well as testing and characterisation of cellular therapeutic products.^111^, ^112^, ^113^, ^114^ Third, standards specify the basic requirements and general considerations for equipment systems and auxiliary materials used in the manufacturing of cells for therapeutic use. They are applicable to any unit operating system used alone or in combination for the manufacturing of cells for therapeutic use, as well as materials used for cell processing and contact with active substances.^115^, ^116^ Fourth, there are also documents that specify not only the general requirements for the competence, impartiality and consistent operation of biobanks, but also the general requirements for the validation and verification of processing methods for all biological materials in biobanks.^117^, ^118^, ^119^ These standards provide guarantee for the healthy development of the SCT industry.

ISO standards related to cell therapy include not only published standards, but also standards under development. Among the 15 standards under development in ISO/TC 276 biotechnology, 5 standards are related to SCT. On the one hand, these standards include requirements for specific types of stem cells. For example, a document named ISO/WD 18162 specifies requirements for the biobanking of human NSCs (hNSCs), including the requirements for the differentiation, culture, characterisation, quality control, storage, thawing, and transport of hNSCs.^120^ It should be noted that this document applies to all organisations performing biobanking of hNSCs used for research and development in the life sciences, and does not apply to hNSCs for the purpose of in vivo application in humans, clinical applications or therapeutic use.^120^ On the other hand, these standards also cover general requirements for cells, including cell morphology analysis, viability analysis, and packing design, as well as data interoperability requirements for stem cell data.^121^, ^122^, ^123^, ^124^ Among them, the requirements for cell morphology analysis and viability analysis are mainly applicable to mammalian cells.^121^, ^123^ Whether it is the published standards or the standards under development, they have made detailed regulations on multiple links in the SCT process. ISO standards are the culmination of the wisdom of experts around the world. They can effectively improve the standardisation of SCT, and are of great significance to the standardisation of the entire industry and human health (Table 2).

**TABLE 2:** SCT‐related standards by ISO.

TC	ISO number	Standards	Scope
ISO/TC 276	ISO 24651:2022	Biotechnology—Biobanking—Requirements for human mesenchymal stromal cells derived from bone marrow	Published
	ISO 24603:2022	Biotechnology—Biobanking—Requirements for human and mouse pluripotent stem cells	Published
	ISO/TS 23565:2021	Biotechnology—Bioprocessing—General requirements and considerations for equipment systems used in the manufacturing of cells for therapeutic use	Published
	ISO 23033:2021	Biotechnology—Analytical methods—General requirements and considerations for the testing and characterization of cellular therapeutic products	Published
	ISO/TS 22859:2022	Biotechnology—Biobanking—Requirements for human mesenchymal stromal cells derived from umbilical cord tissue	Published
	ISO/TR 22758:2020	Biotechnology—Biobanking—Implementation guide for ISO 20387	Published
	ISO 21973:2020	Biotechnology—General requirements for transportation of cells for therapeutic use	Published
	ISO 21899:2020	Biotechnology—Biobanking—General requirements for the validation and verification of processing methods for biological material in biobanks	Published
	ISO 20391−2:2019	Biotechnology—Cell counting—Part 2: Experimental design and statistical analysis to quantify counting method performance	Published
	ISO 20391‐1:2018	Biotechnology—Cell counting—Part 1: General guidance on cell counting methods	Published
	ISO 20387:2018	Biotechnology—Biobanking—General requirements for biobanking	Published
	ISO 20399:2022	Biotechnology—Ancillary materials present during the production of cellular therapeutic products and gene therapy products	Published
	ISO/CD 24479	Biotechnology—Minimum requirements for cellular morphological analysis—Image capture, image processing, and morphometry	Under development
	ISO/FDIS 20404	Biotechnology—Bioprocessing—General requirements for the design of packaging to contain cells for therapeutic use	Under development
	ISO/WD 18162	Biotechnology—Biobanking—Requirements for human neural stem cells derived from pluripotent stem cells	Under development
	ISO/AWI 8934	Biotechnology—General considerations and requirements for cell viability analytical methods—Part 1: Mammalian cells	Under development
	ISO/CD 8472‐1	Biotechnology—Data interoperability for stem cell data—Part 1: Framework	Under development

Abbreviations: AWI, approved work item; CD, committee draft; FDIS, final draft international standard; ISO, International Organization for Standardization; SCT, stem cell‐based therapy; TC, technical committee; TR, technical report; TS, technical specifications; WD, working draft.

### 3.2 SCT‐related standards by international organisations

In order to supplement and update relevant SCT standards in a timely and effective manner, some international organisations have been established to assist in the development of standards. Association for the Advancement of Blood and Biotherapies (AABB, formerly known as the American Association of Blood Banks) is an international, not‐for‐profit organisation representing individuals and institutions involved in the field of transfusion medicine and cellular therapies. AABB sets standards for and accredits facilities that procure, process, store and/or distribute cellular therapy products. The Standards for Cellular Therapy Services outlines the requirements for donor eligibility and collection, processing, storage, and administration for any type of cell therapy product, including UCMSCs services, HSC services, somatic cell services, and cell therapy clinical services.^125^ Similar to AABB, Foundation for the Accreditation of Cellular Therapy (FACT) is well established and mature in the quality management of UCMSCs. FACT is a non‐profit organisation co‐founded by the International Society for Cell and Gene Therapy (ISCT) and the American Society for Transplantation and Cellular Therapy (ASTCT) that sets standards for the high‐quality development of cell therapies. Among the standards established by FACT, those related to SCT mainly include haematopoietic cell therapy standards,^126^, ^127^ cord blood bank standards^128^ and common standards.^129^ The scope of these standards includes HSC, nucleated cells or mononuclear cells from any haematopoietic tissue source (BM, PB, UC and placental blood), cells collected from non‐haematopoietic sources, and cells collected from haematopoietic sources for non‐homologous use.^126^, ^127^, ^128^, ^129^ FACT standards are comprehensive, covering not only all stages before clinical application of cell preparations, such as collection, processing, storage, transportation and management, but also the entire process of clinical application. ISCT not only participated in the formulation of FACT standards, but also independently developed SCT‐related standards, including minimal criteria and immunological characterisation of MSCs.^130^, ^131^ Similar to ISCT, the International Society for Stem Cell Research (ISSCR) has set high standards for SCT as an important international professional organisation engaged in stem cell research. Since 2006, the ISSCR has published four guidelines on stem cell research and clinical translation, promoting scientific inquiry and ethical considerations in the field of stem cells.^132^, ^133^, ^134^, ^135^

In Europe, the European Commission develops the guidance and standards related to cell transplantation through the establishment of the European Directorate for the Quality of Medicines and HealthCare (EDQM). The European Pharmacopoeia (Ph. Eur.) published by EDQM includes a number of quality control standards for cell therapy, such as cell count and viability assays, microbiological examination of cell‐based preparations and production of raw materials for cell‐based therapy products.^136^, ^137^ The official standards published within provide the basis for quality control of SCT. In order to ensure the quality and safety of tissues and cells for the human application, EDQM has also published corresponding guidelines. The latest Guide contains not only general requirements applicable to all tissue establishments and organisations involved in the donation, procurement, testing, processing, preservation, storage and distribution of tissues and cells, but also specific guidelines and requirements for the various tissue and cell types.^138^ However, the SCT‐related standards established in Europe cannot be fully applicable to other countries or regions. In order to achieve harmonisation of standards for drug development and regulation in a wider region, representatives of the organisations in Europe, Japan and the United States discussed and established the International Council for Harmonisation (ICH). The topics of all ICH guidelines are divided into the four categories: Quality Guidelines, Safety Guidelines, Efficacy Guidelines and Multidisciplinary Guidelines. They will ensure the development and registration of safe, effective and high‐quality medicines (Table 3).^139^, ^140^, ^141^, ^142^

**TABLE 3:** SCT‐related standards by international organisations.

Organisations	Year	Standards
AABB	2021	Standards for Cellular Therapy Services, 10th edition
EDQM	2019	European Pharmacopoeia (Ph. Eur.) 10th Edition
	2021	European Pharmacopoeia (Ph. Eur.) 11th Edition
	2022	Guide to the quality and safety of tissues and cells for human application, 5th edition
FACT	2018	Seventh Edition FACT‐JACIE International Standards for Hematopoietic Cellular Therapy Product Collection, Processing and Administration
	2019	Seventh Edition NetCord‐FACT International Standards for Cord Blood Collection, Banking, and Release for Administration
	2019	Second Edition FACT Common Standards for Cellular Therapies
	2021	Eighth Edition FACT‐JACIE International Standards for Hematopoietic Cellular Therapy Product Collection, Processing and Administration
ICH	1997	Q5D. Derivation and Characterisation of Cell Substrates Used for Production of Biotechnological/Biological Products
	1999	Q5A. Viral Safety Evaluation of Biotechnology Products Derived from Cell Lines of Human or Animal Origin
	2011	S6. Preclinical Safety Evaluation of Biotechnology‐Derived Pharmaceuticals
	2022	M10. Bioanalytical Method Validation and Study Sample Analysis
ISCT	2006	Minimal criteria for defining multipotent mesenchymal stromal cells
	2013	Immunological characterisation of multipotent mesenchymal stromal cells
ISSCR	2006	Guidelines for the conduct of human embryonic stem cell research
	2008	Guidelines for the clinical translation of stem cells
	2016	Guidelines for Stem Cell Research and Clinical Translation
	2021	Guidelines for Stem Cell Research and Clinical Translation

Abbreviations: AABB, Association for the Advancement of Blood and Biotherapies; EBMT, European Group for Blood and Marrow Transplantation; EDQM, European Directorate for the Quality of Medicines and Healthcare; FACT, Foundation for the Accreditation of Cellular Therapy; ICH, International Council for Harmonisation; ISCT, International Society for Cell and Gene Therapy; ISSCR, International Society for Stem Cell Research; JACIE, Joint Accreditation Committee ISCT‐Europe and EBMT; Ph. Eur, European Pharmacopoeia; SCT, stem cell‐based therapy.

### 3.3 SCT‐related standards by national organisations

Countries around the world are pursuing global free trade by using a single standard, but it is really not easy to achieve this goal under the current situation. On the one hand, existing international standards can hardly cover the whole process of SCT. On the other hand, the specific conditions of different countries vary significantly, and the same international standard may not apply to all countries. Therefore, on the basis of adopting ISO standards, the national standards bodies of various countries will also adjust and supplement international standards according to their actual needs in order to establish national standards. Founded in 1901, the British Standards Institution (BSI) is the world's first national standards body and a national standards body designated by the UK government. In order to promote the rapid development of stem cell research in the United Kingdom, BSI has publicly released a number of SCT‐related specification documents to help improve the quality and safety of cell products, services and systems. These documents not only cover terms and definitions in regulations relevant to the cell therapy and regenerative medicine industry, but also provide guidance and advice for the development of stem cell‐based regenerative medicine products.^143^, ^144^, ^145^, ^146^ Similarly, German Institute for Standardization (DIN) is an independent platform for standardisation in German and worldwide, representing German interests in international organisations. A standard published by DIN specifies the general requirements for sample containers for storing biological materials in biobanks.^147^ Association Francaise de Normalisation (AFNOR) represents French positions at European and international level. The 2021 survey carried out by AFNOR has revealed that France has secured second place behind Germany when it comes to the number of committees for which it has been entrusted with secretariat duties. In addition to adopting ISO and European standards, AFNOR also publishes purely French standards. As early as 1986, AFNOR published a standard for general terminology in biotechnology.^148^ This standard covered five aspects including terms and definitions, biobanks and biological resource centres, analytical methods, biological processes, data processing and metrology.^148^ Then in 2016, AFNOR published a standard, which was not a cell culture manual, but provided requirements and recommendations to allow the production of cell cultures for virological or serological diagnostics in animal health.^149^

The United States has established a relatively complete standardised stem for cell therapy. In order to achieve cost‐effective, large‐scale, reproducible, and high‐quality cell manufacturing, the National Cell Manufacturing Consortium (NCMC) drafted relevant guidelines for cell manufacturing, clearly listing standards and regulations as one of the key strategic directions for the future development of the United States.^150^, ^151^, ^152^ The American National Standards Institute (ANSI) is the sole representative of ISO in the United States, but it is not a standard development organisation. Rather, American National Standards (ANS) are developed by ANSI through the process of accrediting the procedures of standards development organisations (SDOs) and approving their documents. The SDOs that develop SCT‐related standards mainly include American Society for Testing and Materials (ASTM), Clinical and Laboratory Standards Institute (CLSI) and Parenteral Drug Association (PDA). The standards developed by ASTM include multiple contents of SCT, such as osteogenic differentiation, proliferation, cell counting and characterisation, material adhesion, cell potency determination and so on.^153^, ^154^, ^155^, ^156^, ^157^, ^158^, ^159^, ^160^, ^161^ ASTM has great influence, and its standards are adopted not only by the United States but also by many countries in the world. The standards formulated by CLSI mainly involve the analysis of cellular immunology.^162^, ^163^, ^164^ The PDA has also published the standard for cell therapy control strategies applicable to autologous and allogeneic cell therapy.^165^ In Asia, compared with other neighbouring countries, Japan, South Korea, and China have developed rapidly in the field of SCT in recent years, which is due to their strong government support for regenerative medicine. In Japan, Japanese Industrial Standards Committee (JISC) has published standards on biotechnology vocabulary and containers for cryopreservation of cells.^166^, ^167^ In Korea, Korean Agency for Technology and Standards (KATS) has also published a general guideline of safety test for the cell‐based therapeutic substances.^168^ The national standards published by China are relatively rich in content. These standards include specification requirements for specific cells such as ESCs and porcine PSCs, as well as cell purity, counting, cross‐contamination, and sterility testing.^169^, ^170^, ^171^, ^172^, ^173^, ^174^ There are also many other countries have developed national standards related to SCT, such as France, Sweden, Australia, New Zealand and so on. In addition to national standards, each country may also have group standards, industry standards and local standards, which will supplement national standards in specific areas. With the efforts of all countries in the world, the standardisation of SCT is becoming more and more complete (Table 4).

**TABLE 4:** SCT‐related standards by national organisations.

Countries	Organisations	Year	Standards
America	ANSI	2007	CLSI H42‐A2 Enumeration of Immunologically Defined Cell Populations by Flow Cytometry; Approved Guideline‐Second Edition
		2008	CLSI I/LA29‐A Detection of HLA‐Specific Alloantibody by Flow Cytometry and Solid Phase Assays; Approved Guideline
		2013	CLSI I/LA26‐A2 Performance of Single Cell Immune Response Assays; Approved Guideline—Second Edition
		2017	ASTM F3206‐17 Standard Guide for Assessing Medical Device Cytocompatibility with Delivered Cellular Therapies
		2018	ASTM F3294‐18 Standard Guide for Performing Quantitative Fluorescence Intensity Measurements in Cell‐based Assays with Widefield Epifluorescence Microscopy
		2019	ASTM F3368‐19 Standard Guide for Cell Potency Assays for Cell Therapy and Tissue Engineered Products
		2019	PDA TR 81−2019 Cell‐Based Therapy Control Strategy
		2020	ASTM F2944‐20 Standard Practice for Automated Colony Forming Unit (CFU) Assays—Image Acquisition and Analysis Method for Enumerating and Characterizing Cells and Colonies in Culture
		2021	ASTM F2131‐21 Standard Test Method for In Vitro Biological Activity of Recombinant Human Bone Morphogenetic Protein‐2 (rhBMP‐2) Using the W‐20 Mouse Stromal Cell Line
		2021	ASTM F2997‐21 Standard Practice for Quantification of Calcium Deposits in Osteogenic Culture of Progenitor Cells Using Fluorescent Image Analysis
		2021	ASTM F2998‐14 Guide for Using Fluorescence Microscopy to Quantify the Spread Area of Fixed Cells
		2022	ASTM F3088‐22 Standard Practice for Use of a Centrifugation Method to Quantify/Study Cell‐Material Adhesive Interactions
		2022	ASTM F3106‐22 Standard Guide for in vitro Osteoblast Differentiation Assays
Britain	BSI	2011	PAS 93:2011 Characterization of human cells for clinical applications. Guide
		2012	PAS 84:2012 Cell therapy and regenerative medicine. Glossary
		2012	PAS 83:2012 Developing human cells for clinical applications in the European Union and the United States of America. Guide
		2015	PAS 157:2015 Evaluation of materials of biological origin used in the production of cell‐based medicinal products. Guide
China	SAC	2017	GB/T 35520‐2017 Chemicals—Embryotoxicity—Embryonic stem cell test
		2020	GB/T 38788‐2020 Technical specification for establishment of porcine pluripotent stem cells
		2020	GB/T 39729‐2020 General requirements for measurement of cell purity—Flow cytometry
		2020	GB/T 39730‐2020 General requirements for cell counting—Flow cytometry
		2021	GB/T 40172−2021 General guidance on detection methods of mammalian cell cross‐contamination
		2021	GB/T 40365−2021 General guide for cell sterility testing
France	AFNOR	1986	NF X42‐000 Biotechnology—Vocabulary—General Terms
		2016	NF U47‐200 Animal health analysis methods—Good practice guide for cell cultures
Germany	DIN	2022	DIN 13279 Biotechnology—Requirements for sample containers for storing biological materials in biobanks
Japan	JISC	2020	K3600 Biotechnology—Vocabulary
		2021	K3603 Plastic Vials for Frozen Storage and Ultra Low‐Temperature Preservation
South Korea	KATS	2018	KS P 1600 General guideline of safety test for the cell based therapeutic substances

Abbreviations: AFNOR, French Standardization Association; ANSI, American National Standards Institute; ASTM, American Society for Testing and Materials; BSI, British Standards Institution; CFU, colony forming unit; CLSI, Clinical and Laboratory Standards Institute; DIN, German Institute for Standardization; GB/T, China National Standards; JISC, Japanese Industrial Standards Committee; KATS, Korean Agency for Technology and Standards; NF, Norme Française; PAS, publicly available specification; PDA, parenteral drug association, rhBMP‐2, recombinant human bone morphogenetic protein‐2; SAC, Standardization Administration of China; SCT, stem cell‐based therapy.

## 4 CONCLUSIONS

In the new era of regenerative medicine, the emergence of SCT represents a breakthrough development in biomedical science. Stem cells have made it possible to reverse many incurable diseases through their powerful regeneration and repair ability. After decades of development, SCT has made remarkable achievements in clinical research and market size. The healthy and rapid development of the SCT industry is inseparable from the positive guiding role played by the corresponding standards. SCT standards include three levels: ISO standards, regional organisation standards and national standards. These developed standards have effectively coordinated drug development and regulatory standards between different countries and regions, avoided overlapping standard activities, and improved the application efficiency of stem cell‐related products. However, the standardisation of SCT is still in its infancy. The development direction of standards is scattered, and there is still a lack of a complete standardised system covering all aspects of SCT, which has a negative impact on the clinical application of SCT.

With the continuous deepening and gradual maturity of stem cell related research, the construction of a standardised system for SCT has also been continuously improved. Standardisation and quality control can ensure the safety and effectiveness of SCT. It is believed that SCT will truly realise the transformation from laboratory research to large‐scale clinical practice in the near future.

## AUTHOR CONTRIBUTIONS

All authors contributed to the conception of this work. J Z wrote the manuscript; MR S, JZ W, X L and HG H revised the figure; KZ W, XY L and TZ S revised the tables; ZH L and J L reviewed and revised the full text.

## CONFLICT OF INTEREST STATEMENT

The authors declare they have no conflicts of interest.

## Data Availability

The data that support the findings of this study are available from the corresponding author upon reasonable request.
